# (1′*S*,2*R*,3*R*)-(−)-2-Hydr­oxy-3-morpholino-3-phenyl-*N*-(1′-phenyl­ethyl)propion­amide

**DOI:** 10.1107/S1600536809008198

**Published:** 2009-03-14

**Authors:** Angel Mendoza, David M. Aparicio, Joel L. Terán, Dino Gnecco, Jorge R. Juárez

**Affiliations:** a Centro de Química, ICUAP, Benemérita Universidad Autónoma de Puebla, Puebla, Puebla, Mexico

## Abstract

In the title compound, C_21_H_26_N_2_O_3_, the morpholine ring has a chair conformation and the dihedral angle between the two phenyl rings is 59.0 (3)°. The crystal packing is stabilized by inter­molecular O—H⋯O hydrogen bonds, generating a ribbon structure along the *a* axis. An intra­molecular N—H⋯O contact is also present.

## Related literature

For general background, see: Barbaro *et al.* (1992 ▶); Szymanski *et al.* (2006 ▶); Sheppard *et al.* (2004 ▶); Chen *et al.* (1996 ▶); Concellón *et al.* (2003*a* ▶,*b* ▶); Martín *et al.* (2004 ▶). For related structures, see: Romero *et al.* (2005*a* ▶,*b* ▶). For ring conformation analysis, see: Cremer & Pople (1975 ▶).
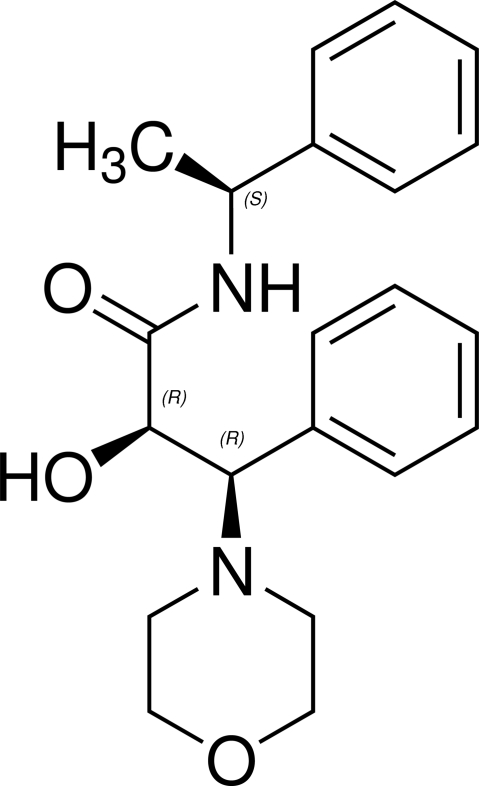


## Experimental

### 

#### Crystal data

C_21_H_26_N_2_O_3_
*M* *_r_* = 354.44
Orthorhombic, 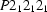

*a* = 6.0010 (17) Å
*b* = 15.659 (3) Å
*c* = 20.746 (4) Å
*V* = 1949.5 (8) Å^3^
*Z* = 4
Mo *K*α radiation
μ = 0.08 mm^−1^
*T* = 293 K
0.72 × 0.28 × 0.16 mm

#### Data collection

Bruker P4 diffractometer
Absorption correction: none
3964 measured reflections
2959 independent reflections
1116 reflections with *I* > 2σ(*I*)
*R*_int_ = 0.055
3 standard reflections every 97 reflections intensity decay: 3%

#### Refinement

*R*[*F* ^2^ > 2σ(*F* ^2^)] = 0.054
*wR*(*F* ^2^) = 0.163
*S* = 0.89
2959 reflections
251 parameters
H atoms treated by a mixture of independent and constrained refinement
Δρ_max_ = 0.24 e Å^−3^
Δρ_min_ = −0.19 e Å^−3^

### 

Data collection: *XSCANS* (Siemens, 1994 ▶); cell refinement: *XSCANS*; data reduction: *XSCANS*; program(s) used to solve structure: *SIR2004* (Burla *et al.*, 2005 ▶); program(s) used to refine structure: *SHELXL97* (Sheldrick, 2008 ▶); molecular graphics: *ORTEP-3 for Windows* (Farrugia, 1997 ▶); software used to prepare material for publication: *WinGX* (Farrugia, 1999 ▶).

## Supplementary Material

Crystal structure: contains datablocks I, global. DOI: 10.1107/S1600536809008198/is2398sup1.cif

Structure factors: contains datablocks I. DOI: 10.1107/S1600536809008198/is2398Isup2.hkl

Additional supplementary materials:  crystallographic information; 3D view; checkCIF report

## Figures and Tables

**Table 1:** Hydrogen-bond geometry (Å, °)

*D*—H⋯*A*	*D*—H	H⋯*A*	*D*⋯*A*	*D*—H⋯*A*
N1—H1*N*⋯O1	1.00 (7)	1.92 (7)	2.569 (5)	120 (5)
O1—H1*O*⋯O2^i^	0.98 (8)	1.80 (8)	2.753 (4)	163 (7)

Symmetry code: (i) 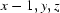.

**Fig. 1.**
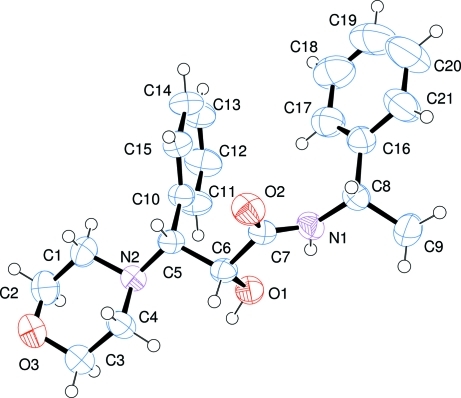
The molecular structure of the title compound, with atom labels and 50% probability displacement ellipsoids for non-H atoms.

**Fig. 2.**
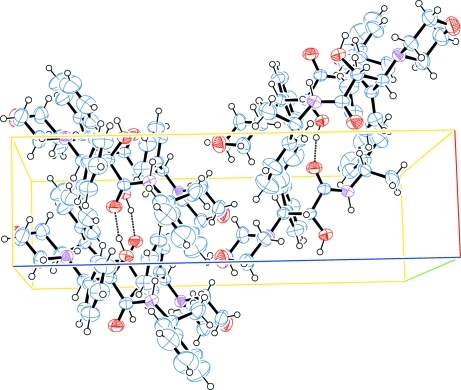
The packing of the title compound, showing molecules connected by O—H···O hydrogen bonds (dashed lines).

## supplementary crystallographic information

### Comment

The stereoselective synthesis of α-hydroxyamides (Barbaro *et al.*, 1992; Szymanski *et al.*, 2006) is an important area in asymmetric synthesis because these kinds of intermediates exhibit a great value as synthetic building block in the synthesis of pharmaceutical, agriculture and medicinal compounds (Sheppard *et al.*, 2004; Chen *et al.*, 1996). Numerous methods have been development through the last 10 years. One of the most relevant methodologies is the opening reaction of epoxyamides (Concellón *et al.*, 2003*a*,*b*; Martín *et al.*, 2004). In this way, we show herein the regiospecific ring opening reaction of epoxyamide derived from (*S*)-(-)-phenyl ethyl amine (Scheme 1). Related structures including (*S*)-(-)-phenyl ethyl amine with different substituents has been previously reported (Romero *et al.*, 2005*a*,*b*).

In the present paper, we report the structure of title compound, (I), which shows a single asymmetric unit with two aromatic rings. The morpholine ring shows an almost perfect chair conformation with a puckering parameters (Cremer & Pople, 1975) (O3—C2—C1—N2—C4—C3) *Q* = 0.572 (4) Å, θ_2_ = 3.6° (4), φ_2_ =343° (10), q_2_ = 0.026 (4) Å and q_3_ = 0.571 (4) Å. In the crystal structure, the molecules are linked by O—H···O hydrogen bonds (Table 1), generating a ribbon structure along the *a* axis.

### Experimental

The compound (I) was obtained by *trans*-diastereoisomeric mixture of epoxyamide in anhydrous EtOH. Then, 1.1 equivalents of morpholine were added. The reaction was stirred over night. Finally, the resultant amino alcohol mixture was separate by column chromatography (AcOEt: Petroleum Ether). The absolute configuration of (1'*S*, 2*R*, 3*R*)-(-)-2-hydroxy-3-morpholin-4-yl-3 -phenyl-*N*-(1'-phenyl-ethyl)-propionamide was established by the structure determination of a (*S*)-(-)-phenyl ethyl amine of known absolute configuration of starting material [C8 in compound (I)]. The H^1^ NMR experiment shows only one disteromeric compound.

### Refinement

H atoms bonded to N and O atoms and all optically relevant were located in a difference map and refined as riding on their parent atoms with *U*_iso_(H) = 1.2*U*_eq_ for N and C—H and *U*_iso_(H) = 1.5*U*_eq_ for O atoms. H atoms linked to C atoms were placed in geometrical idealized positions and refined as riding on their parent atoms, with C—H = 0.93–0.98 Å and with *U*_iso_(H) = 1.2*U*_eq_(C) or 1.5*U*_eq_(methyl C). In the absence of significant anomalous scattering effects, Friedel pairs were merged and the absolute configuration was assigned on the base of synthetic procedure.

### Crystal data

**Table:**

C_21_H_26_N_2_O_3_	*D*_x_ = 1.208 Mg m^−^^3^
*M**_r_* = 354.44	Melting point: 431 K
Orthorhombic, *P*2_1_2_1_2_1_	Mo *K*α radiation, λ = 0.71073 Å
Hall symbol: P 2ac 2ab	Cell parameters from 30 reflections
*a* = 6.0010 (17) Å	θ = 3.9–23.9°
*b* = 15.659 (3) Å	µ = 0.08 mm^−^^1^
*c* = 20.746 (4) Å	*T* = 293 K
*V* = 1949.5 (8) Å^3^	Prism, colorless
*Z* = 4	0.72 × 0.28 × 0.16 mm
*F*(000) = 760	

### Data collection

**Table:**

Bruker P4 diffractometer	*R*_int_ = 0.055
Radiation source: fine-focus sealed tube	θ_max_ = 29.0°, θ_min_ = 1.6°
graphite	*h* = −1→8
2θ/ω scans	*k* = −1→21
3964 measured reflections	*l* = −1→28
2959 independent reflections	3 standard reflections every 97 reflections
1116 reflections with *I* > 2σ(*I*)	intensity decay: 3%

### Refinement

**Table:**

Refinement on *F*^2^	Secondary atom site location: difference Fourier map
Least-squares matrix: full	Hydrogen site location: inferred from neighbouring sites
*R*[*F*^2^ > 2σ(*F*^2^)] = 0.054	H atoms treated by a mixture of independent and constrained refinement
*wR*(*F*^2^) = 0.163	*w* = 1/[σ^2^(*F*_o_^2^) + (0.0711*P*)^2^] where *P* = (*F*_o_^2^ + 2*F*_c_^2^)/3
*S* = 0.89	(Δ/σ)_max_ < 0.001
2959 reflections	Δρ_max_ = 0.24 e Å^−^^3^
251 parameters	Δρ_min_ = −0.19 e Å^−^^3^
0 restraints	Extinction correction: *SHELXL97* (Sheldrick, 2008)
Primary atom site location: structure-invariant direct methods	Extinction coefficient: 0.008 (2)

### Special details

**Table:**

Geometry. All s.u.'s (except the s.u. in the dihedral angle between two l.s. planes) are estimated using the full covariance matrix. The cell s.u.'s are taken into account individually in the estimation of s.u.'s in distances, angles and torsion angles; correlations between s.u.'s in cell parameters are only used when they are defined by crystal symmetry. An approximate (isotropic) treatment of cell s.u.'s is used for estimating s.u.'s involving l.s. planes.
Refinement. Refinement of *F*^2^ against all reflections. The weighted *R*-factor *wR* and goodness of fit *S* are based on *F*^2^, conventional *R*-factors *R* are based on *F*, with *F* set to zero for negative *F*^2^. The threshold expression of *F*^2^ > 2σ(*F*^2^) is used only for calculating *R*-factors(gt) *etc*. and is not relevant to the choice of reflections for refinement. *R*-factors based on *F*^2^ are statistically about twice as large as those based on *F*, and *R*- factors based on all data will be even larger.

### Fractional atomic coordinates and isotropic or equivalent isotropic displacement parameters (Å^2^)

**Table:**

	*x*	*y*	*z*	*U*_iso_*/*U*_eq_	
O1	−0.1563 (5)	0.92034 (19)	0.75127 (13)	0.0567 (8)	
N2	−0.1559 (5)	0.90373 (19)	0.60796 (14)	0.0464 (8)	
O2	0.4127 (5)	0.96834 (18)	0.72379 (13)	0.0598 (8)	
N1	0.2201 (6)	0.9177 (2)	0.81024 (16)	0.0516 (9)	
C10	0.0914 (6)	0.8018 (2)	0.66365 (17)	0.0447 (9)	
C7	0.2354 (6)	0.9439 (2)	0.74924 (19)	0.0445 (9)	
O3	−0.3564 (6)	0.9556 (2)	0.48787 (14)	0.0770 (10)	
C5	0.0430 (7)	0.8947 (2)	0.64872 (18)	0.0449 (9)	
C6	0.0197 (6)	0.9474 (2)	0.71116 (18)	0.0434 (9)	
C4	−0.2007 (7)	0.9933 (2)	0.5920 (2)	0.0554 (11)	
H4A	−0.0697	1.0183	0.5721	0.066*	
H4B	−0.2322	1.0248	0.6312	0.066*	
C8	0.3993 (7)	0.9216 (3)	0.8565 (2)	0.0552 (11)	
C15	0.3024 (8)	0.7691 (2)	0.65361 (19)	0.0535 (10)	
H15	0.4145	0.8045	0.6380	0.064*	
C16	0.5180 (7)	0.8364 (3)	0.86521 (19)	0.0558 (11)	
C11	−0.0702 (7)	0.7484 (3)	0.6885 (2)	0.0610 (11)	
H11	−0.2125	0.7695	0.6964	0.073*	
C17	0.4526 (9)	0.7626 (3)	0.8349 (2)	0.0703 (13)	
H17	0.3292	0.7635	0.8078	0.084*	
C3	−0.3969 (8)	1.0009 (3)	0.5463 (2)	0.0676 (13)	
H3A	−0.5294	0.9782	0.5669	0.081*	
H3B	−0.4235	1.0607	0.5367	0.081*	
C1	−0.1284 (8)	0.8570 (3)	0.54712 (19)	0.0637 (12)	
H1A	−0.1097	0.7967	0.5564	0.076*	
H1B	0.0055	0.8768	0.5257	0.076*	
C2	−0.3229 (9)	0.8683 (3)	0.5030 (2)	0.0765 (15)	
H2A	−0.2973	0.8365	0.4635	0.092*	
H2B	−0.4559	0.8457	0.5233	0.092*	
C13	0.1855 (9)	0.6310 (3)	0.6898 (2)	0.0699 (14)	
H13	0.2158	0.5736	0.6973	0.084*	
C14	0.3492 (8)	0.6836 (3)	0.6666 (2)	0.0638 (12)	
H14	0.4918	0.6623	0.6594	0.077*	
C21	0.7048 (9)	0.8339 (4)	0.9047 (3)	0.0896 (17)	
H21	0.7533	0.8835	0.9250	0.107*	
C9	0.3070 (9)	0.9542 (4)	0.9206 (2)	0.0938 (18)	
H9A	0.2331	1.0078	0.9138	0.141*	
H9B	0.4271	0.9618	0.9506	0.141*	
H9C	0.2027	0.9135	0.9375	0.141*	
C12	−0.0219 (9)	0.6627 (3)	0.7018 (3)	0.0742 (14)	
H12	−0.1316	0.6274	0.7189	0.089*	
C18	0.5674 (12)	0.6867 (3)	0.8440 (3)	0.0958 (18)	
H18	0.5207	0.6370	0.8236	0.115*	
C19	0.7512 (13)	0.6857 (5)	0.8837 (4)	0.119 (2)	
H19	0.8297	0.6351	0.8899	0.143*	
C20	0.8184 (12)	0.7586 (5)	0.9141 (3)	0.122 (2)	
H20	0.9418	0.7574	0.9412	0.147*	
H1O	−0.307 (14)	0.929 (5)	0.735 (4)	0.184*	
H1N	0.059 (12)	0.906 (4)	0.819 (3)	0.147*	
H5	0.180 (12)	0.915 (4)	0.627 (3)	0.147*	
H6	−0.004 (11)	1.009 (4)	0.697 (3)	0.147*	
H8	0.535 (12)	0.960 (4)	0.839 (3)	0.147*	

### Atomic displacement parameters (Å^2^)

**Table:**

	*U*^11^	*U*^22^	*U*^33^	*U*^12^	*U*^13^	*U*^23^
O1	0.0370 (14)	0.0800 (19)	0.0531 (15)	0.0034 (16)	0.0045 (14)	0.0008 (15)
N2	0.0476 (19)	0.0465 (17)	0.0450 (16)	0.0034 (17)	−0.0023 (16)	0.0024 (14)
O2	0.0429 (17)	0.0697 (18)	0.0668 (18)	−0.0070 (15)	0.0011 (15)	0.0061 (15)
N1	0.0388 (18)	0.065 (2)	0.0507 (19)	0.0006 (18)	−0.0067 (17)	−0.0002 (17)
C10	0.041 (2)	0.045 (2)	0.049 (2)	0.003 (2)	0.0017 (19)	0.0029 (17)
C7	0.038 (2)	0.044 (2)	0.051 (2)	0.0032 (18)	−0.001 (2)	−0.0001 (19)
O3	0.091 (3)	0.086 (2)	0.0543 (17)	0.013 (2)	−0.0085 (19)	0.0103 (16)
C5	0.041 (2)	0.046 (2)	0.048 (2)	0.0057 (19)	0.0005 (19)	0.0005 (17)
C6	0.034 (2)	0.047 (2)	0.049 (2)	0.0020 (19)	0.0026 (18)	0.0012 (18)
C4	0.058 (3)	0.050 (2)	0.058 (2)	0.007 (2)	−0.009 (2)	0.008 (2)
C8	0.047 (2)	0.064 (3)	0.055 (2)	0.008 (2)	−0.011 (2)	−0.009 (2)
C15	0.050 (2)	0.052 (2)	0.058 (2)	0.002 (2)	0.005 (2)	−0.001 (2)
C16	0.048 (3)	0.067 (3)	0.053 (2)	0.005 (2)	0.002 (2)	0.003 (2)
C11	0.048 (3)	0.050 (2)	0.085 (3)	0.002 (2)	0.008 (3)	0.009 (2)
C17	0.069 (3)	0.068 (3)	0.074 (3)	0.007 (3)	0.004 (3)	0.008 (3)
C3	0.073 (3)	0.072 (3)	0.058 (2)	0.016 (3)	−0.003 (3)	0.005 (2)
C1	0.080 (3)	0.061 (2)	0.051 (2)	0.007 (3)	−0.003 (3)	−0.010 (2)
C2	0.083 (4)	0.088 (3)	0.058 (3)	0.004 (3)	−0.016 (3)	−0.010 (3)
C13	0.072 (3)	0.047 (2)	0.090 (3)	0.006 (3)	−0.002 (3)	0.008 (2)
C14	0.057 (3)	0.051 (2)	0.084 (3)	0.016 (2)	0.004 (3)	−0.004 (2)
C21	0.075 (4)	0.098 (4)	0.096 (4)	0.018 (4)	−0.030 (3)	0.003 (3)
C9	0.080 (3)	0.134 (5)	0.068 (3)	0.034 (4)	−0.018 (3)	−0.042 (3)
C12	0.063 (3)	0.052 (3)	0.107 (4)	−0.003 (2)	0.003 (3)	0.018 (3)
C18	0.108 (5)	0.063 (3)	0.116 (4)	0.014 (4)	0.016 (4)	0.011 (3)
C19	0.114 (6)	0.094 (5)	0.149 (6)	0.045 (5)	−0.001 (5)	0.025 (5)
C20	0.104 (5)	0.125 (5)	0.137 (6)	0.035 (5)	−0.042 (5)	0.025 (5)

### Geometric parameters (Å, °)

**Table:**

O1—C6	1.410 (5)	C16—C21	1.389 (6)
O1—H1O	0.98 (8)	C11—C12	1.401 (6)
N2—C4	1.466 (5)	C11—H11	0.9300
N2—C1	1.468 (5)	C17—C18	1.387 (7)
N2—C5	1.470 (5)	C17—H17	0.9300
O2—C7	1.248 (4)	C3—H3A	0.9700
N1—C7	1.333 (5)	C3—H3B	0.9700
N1—C8	1.443 (5)	C1—C2	1.494 (7)
N1—H1N	1.00 (7)	C1—H1A	0.9700
C10—C11	1.380 (5)	C1—H1B	0.9700
C10—C15	1.382 (6)	C2—H2A	0.9700
C10—C5	1.514 (5)	C2—H2B	0.9700
C7—C6	1.517 (5)	C13—C12	1.363 (7)
O3—C2	1.416 (5)	C13—C14	1.370 (6)
O3—C3	1.426 (5)	C13—H13	0.9300
C5—C6	1.542 (5)	C14—H14	0.9300
C5—H5	0.99 (7)	C21—C20	1.376 (8)
C6—H6	1.02 (6)	C21—H21	0.9300
C4—C3	1.516 (6)	C9—H9A	0.9600
C4—H4A	0.9700	C9—H9B	0.9600
C4—H4B	0.9700	C9—H9C	0.9600
C8—C16	1.523 (6)	C12—H12	0.9300
C8—C9	1.528 (6)	C18—C19	1.376 (9)
C8—H8	1.08 (7)	C18—H18	0.9300
C15—C14	1.393 (5)	C19—C20	1.365 (9)
C15—H15	0.9300	C19—H19	0.9300
C16—C17	1.373 (6)	C20—H20	0.9300
			
C6—O1—H1O	116 (4)	C16—C17—H17	119.5
C4—N2—C1	107.6 (3)	C18—C17—H17	119.5
C4—N2—C5	111.8 (3)	O3—C3—C4	111.1 (4)
C1—N2—C5	110.8 (3)	O3—C3—H3A	109.4
C7—N1—C8	124.5 (4)	C4—C3—H3A	109.4
C7—N1—H1N	107 (4)	O3—C3—H3B	109.4
C8—N1—H1N	127 (4)	C4—C3—H3B	109.4
C11—C10—C15	118.4 (4)	H3A—C3—H3B	108.0
C11—C10—C5	121.5 (4)	N2—C1—C2	112.3 (4)
C15—C10—C5	120.1 (4)	N2—C1—H1A	109.1
O2—C7—N1	123.7 (4)	C2—C1—H1A	109.1
O2—C7—C6	119.7 (3)	N2—C1—H1B	109.1
N1—C7—C6	116.5 (3)	C2—C1—H1B	109.1
C2—O3—C3	108.5 (3)	H1A—C1—H1B	107.9
N2—C5—C10	111.5 (3)	O3—C2—C1	111.2 (4)
N2—C5—C6	111.0 (3)	O3—C2—H2A	109.4
C10—C5—C6	111.1 (3)	C1—C2—H2A	109.4
N2—C5—H5	112 (4)	O3—C2—H2B	109.4
C10—C5—H5	104 (4)	C1—C2—H2B	109.4
C6—C5—H5	107 (4)	H2A—C2—H2B	108.0
O1—C6—C7	108.7 (3)	C12—C13—C14	120.0 (4)
O1—C6—C5	113.8 (3)	C12—C13—H13	120.0
C7—C6—C5	109.9 (3)	C14—C13—H13	120.0
O1—C6—H6	110 (4)	C13—C14—C15	120.1 (4)
C7—C6—H6	108 (4)	C13—C14—H14	120.0
C5—C6—H6	106 (4)	C15—C14—H14	120.0
N2—C4—C3	111.0 (3)	C20—C21—C16	120.5 (6)
N2—C4—H4A	109.4	C20—C21—H21	119.8
C3—C4—H4A	109.4	C16—C21—H21	119.8
N2—C4—H4B	109.4	C8—C9—H9A	109.5
C3—C4—H4B	109.4	C8—C9—H9B	109.5
H4A—C4—H4B	108.0	H9A—C9—H9B	109.5
N1—C8—C16	113.0 (3)	C8—C9—H9C	109.5
N1—C8—C9	108.8 (3)	H9A—C9—H9C	109.5
C16—C8—C9	111.1 (4)	H9B—C9—H9C	109.5
N1—C8—H8	112 (3)	C13—C12—C11	120.1 (5)
C16—C8—H8	100 (3)	C13—C12—H12	119.9
C9—C8—H8	112 (3)	C11—C12—H12	119.9
C10—C15—C14	120.8 (4)	C19—C18—C17	119.4 (6)
C10—C15—H15	119.6	C19—C18—H18	120.3
C14—C15—H15	119.6	C17—C18—H18	120.3
C17—C16—C21	118.5 (5)	C20—C19—C18	120.2 (6)
C17—C16—C8	123.3 (4)	C20—C19—H19	119.9
C21—C16—C8	118.1 (4)	C18—C19—H19	119.9
C10—C11—C12	120.6 (4)	C19—C20—C21	120.4 (6)
C10—C11—H11	119.7	C19—C20—H20	119.8
C12—C11—H11	119.7	C21—C20—H20	119.8
C16—C17—C18	121.1 (5)		
			
C8—N1—C7—O2	6.1 (6)	N1—C8—C16—C17	−3.5 (6)
C8—N1—C7—C6	−170.8 (3)	C9—C8—C16—C17	119.1 (5)
C4—N2—C5—C10	−178.6 (3)	N1—C8—C16—C21	175.0 (4)
C1—N2—C5—C10	−58.5 (4)	C9—C8—C16—C21	−62.5 (5)
C4—N2—C5—C6	57.0 (4)	C15—C10—C11—C12	−1.3 (6)
C1—N2—C5—C6	177.1 (3)	C5—C10—C11—C12	179.9 (4)
C11—C10—C5—N2	−51.9 (5)	C21—C16—C17—C18	1.0 (7)
C15—C10—C5—N2	129.3 (4)	C8—C16—C17—C18	179.5 (4)
C11—C10—C5—C6	72.4 (5)	C2—O3—C3—C4	60.3 (5)
C15—C10—C5—C6	−106.4 (4)	N2—C4—C3—O3	−59.3 (5)
O2—C7—C6—O1	−177.3 (3)	C4—N2—C1—C2	−54.6 (5)
N1—C7—C6—O1	−0.2 (4)	C5—N2—C1—C2	−177.1 (4)
O2—C7—C6—C5	57.6 (5)	C3—O3—C2—C1	−59.9 (5)
N1—C7—C6—C5	−125.4 (3)	N2—C1—C2—O3	59.0 (5)
N2—C5—C6—O1	63.3 (4)	C12—C13—C14—C15	−1.7 (7)
C10—C5—C6—O1	−61.3 (4)	C10—C15—C14—C13	−0.3 (7)
N2—C5—C6—C7	−174.5 (3)	C17—C16—C21—C20	−1.3 (8)
C10—C5—C6—C7	60.9 (4)	C8—C16—C21—C20	−179.8 (6)
C1—N2—C4—C3	54.3 (5)	C14—C13—C12—C11	2.1 (8)
C5—N2—C4—C3	176.2 (3)	C10—C11—C12—C13	−0.6 (7)
C7—N1—C8—C16	−99.7 (5)	C16—C17—C18—C19	−0.6 (8)
C7—N1—C8—C9	136.4 (4)	C17—C18—C19—C20	0.4 (10)
C11—C10—C15—C14	1.7 (6)	C18—C19—C20—C21	−0.7 (11)
C5—C10—C15—C14	−179.4 (4)	C16—C21—C20—C19	1.1 (10)

### Hydrogen-bond geometry (Å, °)

**Table:**

*D*—H···*A*	*D*—H	H···*A*	*D*···*A*	*D*—H···*A*
N1—H1N···O1	1.00 (7)	1.92 (7)	2.569 (5)	120 (5)
O1—H1O···O2^i^	0.98 (8)	1.80 (8)	2.753 (4)	163 (7)

Symmetry codes: (i) *x*−1, *y*, *z*.
